# Can Thumbs UP: The Prevention arm of the Canadian Consortium on Neurodegeneration in Aging (CCNA)

**DOI:** 10.1002/alz70858_100229

**Published:** 2025-12-25

**Authors:** Sylvie Belleville, Nicole D. Anderson, Paul W.H. Brewster, Andrew Lim, Manuel Montero‐Odasso, Haakon B. Nygaard, Howard H. Feldman, Howard Chertkow, CCNA‐CAN‐THUMBS UP Study Group

**Affiliations:** ^1^ Institut Universitaire de Gériatrie de Montréal, Montréal, QC, Canada; ^2^ Rotman Research Institute, Toronto, ON, Canada; ^3^ University of Victoria, Victoria, BC, Canada; ^4^ Sunnybrook Research Institute, Toronto, ON, Canada; ^5^ Schulich School of Medicine & Dentistry, Division of Geriatric Medicine, Western University, London, ON, Canada; ^6^ University of British Columbia, Vancouver, BC, Canada; ^7^ University of California San Diego, La Jolla, CA, USA; ^8^ Alzheimer's Disease Cooperative Study (ADCS), University of California San Diego, La Jolla, CA, USA; ^9^ Rotman Research Institute, Baycrest Health Sciences, Toronto, ON, Canada

## Abstract

**Background:**

In 2019 Canadian dementia researchers launched the “The Canadian Therapeutic Platform Trial for Multidomain Interventions to Prevent Dementia” (CAN‐THUMBS UP; CTU), a national program to advance dementia prevention in Canada and globally. The initiative responded to findings demonstrating that lifestyle interventions could improve cognitive decline and to the Lancet Commission's report that over 40% of dementia cases might be prevented through risk factor mitigation.

**Method:**

A major focus of CTU was developing and validating the Brain Health PRO Program, a 45‐week, bilingual, web‐based educational intervention addressing modifiable risk factors. The program consists of 181 ten‐minute chapters delivered incrementally on a weekly basis. It was designed to foster engagement and convey the best available evidence for lifestyle changes. A total of 345 participants self‐registered, completed initial questionnaires and gained access to the intervention. Dementia literacy, the primary outcome, was measured with the Alzheimer's Disease Knowledge Scale. Secondary outcomes included self‐efficacy (General Self‐Efficacy Scale), attitude toward dementia, user experience (System Usability Scale and Technology Acceptance Model Questionnaire), lifestyle, standard cognitive testing, smartphone‐based cognitive assessment, 24‐hour continuous mobility and sleep measures using actigraphy and sleep EEG wearables (Feldman, Belleville et al. 2023).

**Result:**

The validation study successfully achieved its primary outcome of improving dementia literacy. Positive effects were also observed for secondary outcomes, including self‐efficacy and user satisfaction, with preliminary evidence suggesting a reduction in modifiable risk factors. Three additional studies leveraged the CTU infrastructure: SYNERGIC@Home, assessing the feasibility of a home‐based intervention to improve gait and cognition; SYNERGIC‐2, a randomized controlled trial testing a 12‐month home‐based personalized multidomain intervention in individuals at risk for dementia, using Brain Health PRO as a control condition; and an implementation sub‐study conducted in collaboration with the Federation of Quebec Alzheimer Societies.

**Conclusion:**

CTU reflects a vibrant, collaborative effort of over 100 investigators, staff, citizen advisors, and partners. Leveraging CCNA's team structure, CTU incorporated contributions in study design, recruitment, technology, program evaluation and participatory research. Larger studies are underway within CCNA to confirm the efficacy of Brain Health PRO in mitigating dementia risk and to examine its implementation in diverse settings.

